# Hypothesis: hematogenous metastatic cancer cells of solid tumors may disguise themselves as memory macrophages for metastasis

**DOI:** 10.3389/fonc.2024.1412296

**Published:** 2024-07-05

**Authors:** Chuo Jiang, Jiaxi Wu

**Affiliations:** ^1^ School of Life Sciences, Shanghai University, Shanghai, China; ^2^ Central Laboratories, Shanghai Clinical Research Center Xuhui Central Hospital, Chinese Academy of Sciences, Shanghai, China; ^3^ Office of Industrial Cooperation, Shanghai Institute of Nutrition and Health, Chinese Academy of Sciences, Shanghai, China

**Keywords:** metastasis, macrophage, trained macrophage, memory macrophage, fusion, hybridization, spontaneous regression, Coley’s toxin

## Abstract

German pathologist Otto Aichel suggested, a century ago, that the cancer cell acquired its metastatic property from a leukocyte via cell-cell fusion. Since then, several revised versions of this theory have been proposed. Most of the proposals attribute the generation of the metastatic cancer cell to the fusion between a primary cancer cell and a macrophage. However, these theories have not addressed several issues, such as dormancy and stem cell-like self-renewal, of the metastatic cancer cell. On the other hand, recent studies have found that, like T- and B-/plasma cells, macrophages can also be categorized into naïve, effector, and memory/trained macrophages. As a memory/trained macrophage can enter dormancy/quiescence, be awakened from the dormancy/quiescence by acquainted primers, and re-populate via stem cell-like self-renewal, we, therefore, further specify that the macrophage fusing with the cancer cell and contributing to metastasis, belongs with the memory/trained macrophage, not other subtypes of macrophages. The current theory can explain many puzzling clinical features of cancer, including the paradoxal effects (recurrence vs. regression) of microbes on tumors, “spontaneous” and Coley’s toxin-induced tumor regression, anticancer activities of β-blockers and anti-inflammatory/anti-immune/antibiotic drugs, oncotaxis, surgery- and trauma-promoted metastasis, and impact of microbiota on tumors. Potential therapeutic strategies, such as Coley’s toxin-like preparations, are proposed. This is the last article of our trilogy on carcinogenesis theories.

## 1 Introduction

As a hallmark of cancer, metastasis is conceived as a unique intrinsic property of the primary cancer cell (priCaC) of solid tumors. On the other hand, German pathologist Otto Aichel proposed, more than a century ago, that the cancer cell acquired the metastatic ability following its fusion with the macrophage (Mφ). Since then, a number of revised versions of this theory have repeatedly been brought forward (1–8). These revised versions have argued that, via fusion with the Mφ, the metastatic cancer cell (metCaC) acquires cell migration-associated proteins from the Mφ, which confer its ability to metastasize (or migrate) to distant organs like the Mφ. However, most of the revised theories have not discussed other important biological properties, such as dormancy and stem cell-like self-renewal (9), of the metCaC.

Recently, a subpopulation of Mφs have been found to have immune memory, just like memory adaptive immune lymphocytes. These so called trained or memory Mφs (memMφs) can enter dormancy/quiescence and repopulate via self-renewal like the stem cell (10). Considering that memMφs and metCaCs share the same biological behaviors, as well as that metCaCs expresse Mφ-specific and memMφ-specific surface markers (**Table 1**), we, therefore, take Otto Aichel’s theory a step further and propose that metCaCs camouflage themselves as memMφs to metastasize from the inflammatory primary tumor site to distant organs following their fusion with memMφ. We present evidence to support our theory (Section 2) and suggest a model describing how metCaCs are generated (Section 3). We then use the theory to interpret a number of puzzling clinical presentations of metCaCs (Section 4), as well as to suggest potential therapeutic strategies to treat cancer patients (Section 5). We also raise several issues related to the theory (Section 6).

**Table 1:** Examples of immune cell surface markers expressed by metastatic cancer cells,.

Immune cell surface markers expressed by metastatic cancer cells	Immune cells	Reference
CD45RO^+^	Memory lymphocyte	(11)
CD204, CD206, CD163	M2 macrophage	(1)
Pattern recognition receptors	Memory macrophage	(12, 13)

A tremendously number of pioneering and inspiring studies have been conducted on tumor hybrid cells (THCs), including myeloid and tumor hybrid cells (9, 14). The current article addresses only one type of the THCs, the hybrid between the memMφ and the tumor cell. Additionally, as cancer is a complex disease, the current hypothesis is only intended to address the hematological metastatic cancer cell of solid tumors that fuses or hybridizes with Mφs/memMφs, acquires biochemical, biological, and immunological properties from the Mφs/memMφs, and can metastasize/migrate to distant organs like the Mφ/memMφs.

**Box 1** lists the terms and their connotations conferred specifically by this article. **Box 1** also includes the abbreviations used by this article. Specifically, we refer the “priCaC-memMφ hybrid” as the “MEMORY macrophage-like tumor hybrid cell (memMφ-like THC)” which is equivalent to the “metCaC”. The term of “memMφ-like THC” and the term of “metCaC” are used interchangeably throughout this article. Additionally, we refer the hybrid between the priCac and any Mφ subtypes as the “macrophage-like tumor hybrid cell (Mφ-like THC)”.

Box 1 **Glossary and abbreviations**

**1 Glossary**

• Macrophage-like tumor hybrid cell: refers to the hybrid between a primary cancer cell and a macrophage.

• Memory macrophage-like tumor hybrid cell: refers to the hybrid between a primary cancer cell and a memory/trained macrophages. Here, the “memory macrophage-like tumor hybrid cell” is equivalent to the “metastatic cancer cell” and these two terms are used interchangeably.

• Metastatic cancer cell, hematogenous: refers to the cancer cell that fulfills the entire metastatic process, including dormancy, exit from dormancy, and stem-like self-renewal. Here, the “metastatic cancer cell” is equivalent to the “memory macrophage-like tumor hybrid cell” and these two terms are used interchangeably.

• Primary cancer cell: refers to the cancer cell of origin that has not been hybridized with the macrophage or cell of another type.

• Primary tumor site: refers to the site where the primary tumor is developed. Unlike the primary cancer cell which is the cancer cell of origin, the primary tumor site contains not only the primary cancer cell but also other types of cancerous cells such as the macrophage-like tumor hybrid cell and memory macrophage-like tumor hybrid cell, as well as inflammatory and stromal cells.

**2 Abbreviations**

- CTC: circulating tumor cell
- DAMP: damage-associated molecular pattern
- DTC: disseminated tumor cell
- HSC: hematopoietic stem cell
- HSPC: hematopoietic stem and progenitor cell
- ICD: Immunogenic cell death
- Mφ: macrophage
- MAMP: pathogen- or microbe-associated molecular pattern
- memMφ: memory or trained macrophage
- metCaC: hematogenous metastatic cancer cell
- NSAID: non-steroidal anti-inflammatory drug
- PBMC: peripheral blood mononuclear cell
- PCD: programmed cell death
- priCaC: primary cancer cell
- PRR: pattern recognition receptor
- RCC: renal cell carcinoma
- THC: tumor hybrid cell

## 2 The sharing biological properties between metastatic cancer cells and memory macrophages

The biochemical and biological resemblances (such as cell surface markers, local invasive growth, intravasation, immune evasion, extravasation, organotropism, and niches) between metastatic metCaCs (15–25) and migratory memMφs and their lineage-specific ancestor hematopoietic stem cells (HSCs), progenitors, and precursors located at both medullary and extra-medullary hematopoietic niches and inflammatory sites (21–24, 26–34), has been discussed extensively throughout the literature including the articles on the fusion theory that we cite (1–8) and, due to word limitations, they are not re-iterated here. **Table 1** also enumerates the surface markers acquired by priCaCs that are typically expressed by Mφs, memMφs, lymphocytes, and memory lymphocytes. Readers who are interested in the detail may refer to the references cited in this article.

It has long been known that only a very limited number of disseminated tumor cells (DTCs) enter dormancy as metCaCs (23), that the dormant metCaCs often release a limited number of cells into the blood circulation (i.e., clinically observed “immunological dormancy”) (35), that the dormant metCaCs are awakened from the dormancy by traumatic and infectious stresses (which have been proposed to be, presumably, caused by reduced immunity, transfusion, etc), (36, 37), and that the awakened metCaCs possess the stem cell-like self-renewal property to re-populate themselves. Likewise, memMφs are also a small subpopulation of Mφs, enter dormancy at a reservoir organ following the resolution of an inflammation (38–41), release sentinels into the blood circulation to patrol for pathogens and damaged autologous cells (42), are awakened from the dormancy by acquainted infectious pathogens and/or damaged cells, and act as stem cell-like cells to self-renew to expand their population for clearing the pathogens or damaged cells. Together with the biochemical and biological resemblances between migratory memMφs and metastatic metCaCs (15–34), including the shared surface markers (**Table 1**), these observations strongly suggest that the metCaC is primarily a hybrid of the priCaC and the memMφ.

As a hybrid of the priCaC and memMφ, the memMφ-like THC/metCaC inherits cellular features of both parental cells of origin and, therefore, possess the mutator phenotype like the priCaC, migrate across the body like a monocyte/Mφ (43–45), and enter dormancy like the memMφ. It should be noted that so far evidence only supports the migration of monocytes from the bone marrow to the inflammatory periphery but not the reversed process, i.e., the migration of conventional Mφs from the periphery to the bone (10). (Please refer to Section 6.1 for further discussion.) Nevertheless, the priCaC acquires the property to metastasize/migrate from the periphery to distant organs following its hybridization with the Mφ (1–9, 46, 47).

Consistent with the current hypothesis, memMφ-like THCs/metCaCs often metastasize to distant organs, such as the lungs, liver, brain, and bone that are highly populated with morphologically unique tissue-resident Mφs such as alveolar Mφs, osteoclasts, Kupffer cells, and microglia. Likewise, memMφ-like THCs/metCaCs and memMφs also share reprogrammed metabolism, for example, glycolytic metabolism, and epigenetic profiles (48, 49), as well as stem-cell signaling pathways such as Notch and WNT (50, 51). Actually, the clinical imaging of metastatic tumors is almost the same as that of extramedullary hematopoieses (52). Moreover, several studies showed that metCaCs acquire the stem cell-like property from Mφs as a result of fusion (53, 54). Additionally, priCaCs were also found to receive immune regulatory molecules, such as immune suppressive molecules CTLA4 and Tim3, from immune cells via cellular events such as trogocytosis (55). Consistently, a benign tumor was converted into a malignant one following the fusion of its cells with leukocytes (46). Supportive pieces of evidence have also been provided experimentally. Fusing non-metastatic cancer cells with normal leukocytes or fusing cancer stem cells with monocytes confers the feature of metastasis to the non-metastaic parental cancer cells (8, 9, 47).

## 3 Model for the generation of the memory macrophage-like tumor hybrid cell/metastatic cancer cell

### 3.1 Model for the generation of memory macrophage-like hybrid cells/metastatic cancer cells

To further support those earlier theories and our theory that have been discussed above (Section 2), particular our view that the metCaC is a hybrid between the priCaC and the memMφ, not just any Mφs, we propose the following model to interpret how the hybrid is generated. This model is founded on recent advances in several research fields. (Please refer to **Box 2** for a brief introduction.) The theory is also founded on the similarity between virus-induced (non-sterile) and sterile (including priCaC-induced) inflammatory processes (44, 91, 92), as well as the shared immunological principle between memMφs and memory T cells (**Box 2** Item 17).

Box 2 **A brief introduction to recent advances in several research fields**

**(1) Ontogeny and subsets of macrophages**

Many peripheral organs have two or more ontogenically distinctive Mφs populations (56, 57), including tissue-resident Mφs, recruited Mφs, and, sometimes, interstitial Mφs. The recruited Mφs are from the bone marrow via the blood circulation afterbirth, whereas the tissue-resident Mφs are ontogenically colonized from embryonic organs, such as the liver, at early embryonic stages (56, 57). The recruited Mφs can differentiate into the tissue-resident Mφs to replenish the vacant Mφ niche when the original tissue-resident Mφs are depleted during events such as an inflammation (56, 57).

**(2) Plasticity and states of macrophages**

Additional to the well-known M1 and M2 Mφ, a spectrum of polarized states of Mφs have been identified, which are induced by a variety of primers such as MAMPs from microbes and DAMPs from damaged host cells (56, 57). Moreover, a Mφ can be of both M1 and M2 states simultaneously, showing both pro- and anti-inflammatory activities (57).

**(3) Subtypes of macrophages**

Like lymphocytes, Mφs are categorized into memMφs and effector Mφs, as well as naïve monocytic Mφs (i.e., monocytes) (58). The memMφs can further be classified as tissue-resident memMφs, “central”/distant memMφs, etc. (38). The “central”/distant memMφs can be recruited by an inflammatory organ and differentiate into the tissue-resident Mφs to replenish the vacant Mφ niche when the original tissue-resident Mφs are depleted (56, 57).

**(4) Memory hematopoietic stem cells and memory cells of other tissue/organs**

Additional to lymphocytes and Mφs, HSPCs of multiple lineages (59), as well as many parenchymal cells of differentiated tissue (60), can also be primed to have memory.

**(5) Medullary (bone marrow) and extra-medullary (peripheral organs) hematopoiesis**

Hematopoiesis primarily occurs at specific niches for HSPCs within either the bone marrow (called medullary hematopoiesis) or peripheral organs (called extra-medullary hematopoiesis) (61). Under stress conditions such as trauma and infection, priming MAMPs and priming DAMPs are respectively released from microbes and damaged host cells (Item 12 of this Box). The MAMPs and/or DAMPs bind specifically to their cognate receptors, PRRs, on the dormant memory HSPCs, awakening the latter. The lineage-biased emergency hematopoiesis involving memMφs or other myeloid lineage cells can be quickly induced by the MAMPs/DAMPs (62, 63). However, long-term exposure to MAPMs/DAMPs or exposure to the high-dose of MAMPs/DAMPs can lead to impaired hematopoiesis (62, 63). For example, recurrent and chronic infections lead to the depletion of HSPCs (62).

**(6) Medullary and extramedullary niches for hematopoietic stem and progenitor cells and niche-to-niche migration of HSPCs**

HSPCs, including memory HSPCs, can be activated and egress out of the bone marrow and enter into the blood circulation. They migrate or are recruited to inflammatory peripheral organs (41, 59). *Vice versa*, primed immune cells, including memory immune cells, can also migrate from the inflammatory peripheral organs to lodge at the bone marrow (7). Cancerous cells can also reciprocally migrate between the peripheral organs and the bone marrow (7).

**(7) Medullary and extramedullary niches for macrophages or dormant macrophages**

A number of organs, such as the liver, lungs, bone and brain, have niches specifically designated to Mφs (56, 57, 64).

**(8) Niche-to-niche migration of monocytes and macrophages**

Under steady-state conditions, central memMφs at the bone marrow and tissue-resident memMφs of various subsets (or pools) at peripheral organs (Items 1 and 3 of this Box), are often localized at their designated sites (Item 7 of this Box). The moncytes/Mφs located at both bone marrow and peripheral organs can self-renew to perpetuate their populations (56, 57). However, when the peripheral organs experience a severe sterile and/or non-sterile inflammation, the tissue-resident Mφs can be consumed and even depleted (Item 1 of this Box). Under such a condition, the central moncytes, which have previously been primed by the same primer, from the bone marrow are recruited to the diseased organ and differentiation into Mφs (56–58, 65).

Once the inflammation is resolved (Item 9 of this Box), the recruited monocytes/Mφs can be phenotypically switched to the tissue-resident Mφs to replenish the depleted Mφs niche and restock the tissue-resident Mφs, probably in the form of the tissue-resident memMφs (66, 67).

**(9) Sterile and non-sterile inflammatory processes, their resolution phases and generation of memory immune cells at barrier tissue**

Conventional inflammations, both sterile and non-sterile ones, are composed of three phases: initiation/activation, expansion, and resolution. Damaged host cells and microbes can respectively release DAMPs and MAMPs (68). The damaged host cells and microbes can also be phagocytosed, further secreting the MAMPs and DAMPs. The MAMPs and DAMPs prime various types of tissue-resident immune cells, including tissue-resident Mφs, as well as awaken and recruit various types of memory immune cells from the bone marrow and other peripheral organs (69). With the clearance of the damaged host cells and microbes, the inflammatory response proceeds into the reparative resolution phase. Memory immune cells, such as tissue-resident memMφs and tissue-resident memory T cells, as well as “central”/distant memMφs and “central”/distant memory T cells, are generated (39). Cancerous cells can be generated during the reparative resolution phage of an inflammation and the generation is mediated by leukocytes (46, 70).

**(10) Barrier tissues and commensals and pathogenic microbes at the barrier tissues, and their contribution to innate immune memory**

The tissue/organ that is directly exposed to the external environment, such as the skin and the gut, is called the barrier tissue/organ or, simply, the barrier (60). Commensals and pathogenic microbes are located at the barrier. Damage of the barrier exposes the microbes to innate and adaptive immune cells, causing inflammatory and immune responses. As a result, memory immune cells, such as tissue-resident memMφs and tissue-resident memory T cells, can be primed and generated (Items 9 and 17 of this Box).

**(11) Cancer-associated microbes**

There are generally two sources of microbes that are associated with cancer: intratumoral microbes (3, 71) and microbes at the compromised barrier tissue/organ (Item 10 of this Box). Under inflammatory conditions, they respectively release MAMPs and DAMPs. The MAMPs/DAMPs can prime and convert MAMP/DAMP-naïve monocytes and Mφs into their memory counterparts (Item 12 of this Box), contributing to innate immune memory (72, 73).

**(12) Damage-associated molecular patterns (DAMPs) and microbe/pathogen-associated molecular patterns (MAMPs) and MAMP/DAMP-specific memory macrophages**

Lysed commensal and pathogenic microbes and damaged host cells can respectively release MAMPs and DAMPs (74). The MAMPs and DAMPs can also be secreted by Mφs that have phagocytosed the microbes and damaged host cells. The MAMPs/DAMPs can prime Mφs, generating memMφs (74). Like memory T and B lymphocytes, the primed memMφs are primer-specific (74). However, the specificity is not as strictly as the T and B lymphocytes. The MAMPs and DAMPs can be classified into several categories, such as double-stranded DNA, lipopolysaccharides and lipopeptides, according to the chemical structure (74). Each category of the MAMPs/DAMPs can bind to their specific cognate receptors, PRRs, on the Mφs, activating specific downstream signaling pathways (68, 74). For example, double stranded DNA binds to TLR9 receptor whereas bacterial lipopolysaccharides and lipopeptides/lipoproteins respectively interact with TLR4 and TLR2 (68, 74). As a result, the MAMPs/DAMPs of the same category of the chemical structure can activate the same memMφs, often irrespective of species, strains or types of the microbes/damaged host cells with which the MAMPs/DAMPs belong (74).

Additional to being primers, the MAMPs and DAMPs can also awaken specific dormant memMφs that have previously been primed by the same MAMPs/DAMPs (74).

**(13) Immune specificity, including compartmentalization, of memory macrophages and their effector progeny**

Like adaptive antibody-generating B/plasma cells and T-cell receptor (TCR)-expressing T cells, MAPMP/DAMP-primed Mφs/memMφs are specific to the priming MAPMs/DAMPs (Item 12 of this **Box 2**). However, as discussed above (Item 12 of this **Box 2**), the specificity of the Mφs/memMφs is not as strictly as the adaptive immune cells (75). The specificity is conferred by the specific binding of the MAPMPs/DAMPs to their specific cognate PRRs (74) which is chemical structure-dependent, often irrespective of the species, strains or types of the microbes/damaged host cells that release the MAPMPs/DAMPs (Item 12 of this **Box 2**).

The specificity between the biding of the priming MAMPs/DAMPs to the PRRs also leads to the phenomenon of “compartmentalization” (76). The species, strains and quantity of the commensal and/or pathogenic microbes at different anatomic sites of the body (i.e., the local microbiome), even within the same organ, are distinct. Likewise, types and cellular states of the cells at different anatomic sites are distinct as well. Therefore, the anatomic site-specific MAMPs and DAMPs lead to the generation of anatomic site-specific tissue-resident Mφs/memMφs. This phenomenon is called “compartmentalization” (76), which might be the underlying reason that eczema and immune colitis often repeatedly occur at the same region or segment of the organ (77). Consistent with the anatomic site-specificity of the memMφs, Mφs at different anatomic sites respond differently to the same type of the sterile inflammation (78).

**(14) DNA repair-defective primary cancer cells**

Cancer has been proposed to be a DNA repair-defective disease (79, 80). The proposal is primarily founded on three pieces of evidence. Genetically, congenital syndromes that predispose to cancer, are always defective in certain genes encoding DNA repair proteins; biochemically, cancer cells are always defective in certain DNA repair pathways; and biologically, cancer cells express the mutator phenotype, including elevated single nucleotide alterations and chromosome structure aberrations (i.e., genomic instability), which suggests that the DNA has been mis-repaired, indicative of DNA repair defects.

DNA repair-defective cells are prone to DNA damagers such as intracellular free radicals, DNA-damaging anticancer drugs and ionizing radiotherapy, etc. Because the generation of T-cell receptors and antibodies requires normal DNA repair (such non-homologous end-joining and mismatch repair), patients having DNA-repair defects cannot elicit the adaptive immunity in the same way as a normal individual (79, 80).

**(15) Programmed cell death, immunogenic cell death and immunological memory**

Cells can undergo programmed cell death (PCD) and senescence, as a result of DNA damage and other stresses (81). The cells undergoing the PCD and senescence may release cell debris and cellular molecules (such as MAMPs/DAMPs and cytokines) to cause inflammatory and immune responses. Immunogenic cell death (ICD) is one form of the PCD. The ICD can be induced by MAMPs and DAMPs (72). ICD is believed to be associated with the immunological memory (72, 73). Cancer cells can also undergo ICD (82, 83, 91).

**(16) Protective immunity, depletion, exhaustion, tolerance, and other immune states of primed macrophages**

Not all priming of Mφs by MAMPs/DAMPs can lead to the normal protective immunity of the Mφs. Depending on the dose and mode of the MAMPs/DAMPs, the Mφs can also be induced to enter other immune states such as depletion, tolerance, exhaustion, anergy, paralysis, etc. (84–86). Some of these immune states described by different authors seem to refer to the same or overlapping immune states (86). For simplicity, as well as the purpose of this article, we refer the so-called depletion of Mφs as a QUANTITATIVE depletion of the Mφs.

**(17) Memory T cell and its properties**

Memory T cells can be categorized into several subtypes such as resident memory T cells and central memory T cells (87). The tissue-resident memory T cells remain at the inflammatory site following the resolution of an inflammation (40). The central memory T cells mobilize to distant niches located at the secondary lymphoid tissue (such as lymph node), bone marrow, and other organs (41). At the steady state, most of these memory T cells remain dormant (87) and a few patrol across the body via the blood circulation (88). However, under stress conditions, the dormant memory T cells can be awakened and recruited to the inflammatory site (88, 89). Some of the awakened memory T cells possess stem cell-like property and, by self-renewal (87), can perpetuate their population and elicit a protective immune response. However, the awakened memory T cells and their effector progeny can also be induced into other immune states such as exhaustion in chronic viral infection and cancer (40, 90).

**Figure 1:**
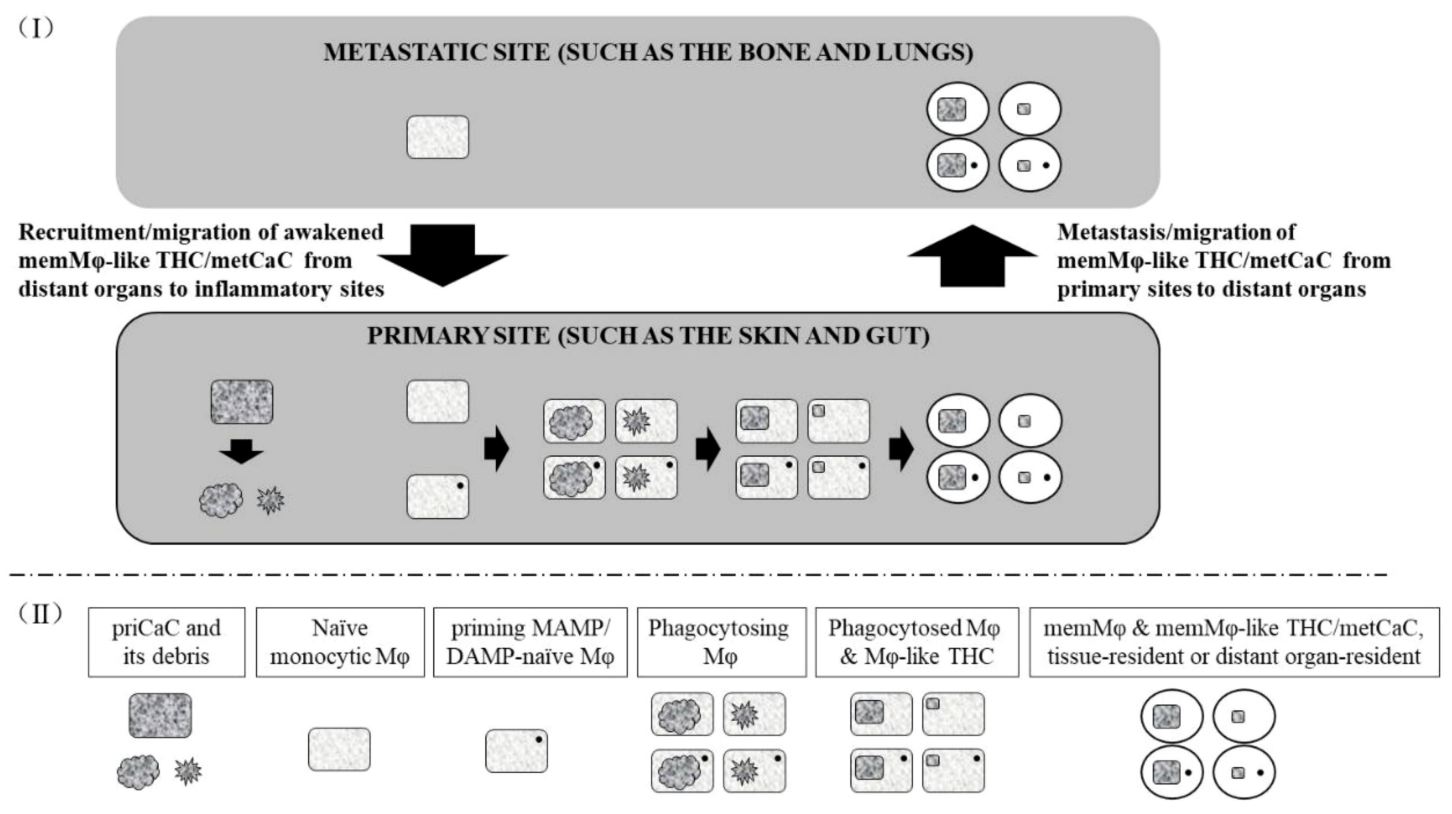
Model for the generation of the memory macrophage-like tumor hybrid cell/metastatic cancer cell. (I) The primary cancer cell (priCaC) undergoes programmed cell death or senescence, initiating an inflammation. The naïve monocytic macrophages (Mφ) and priming MAMP/DAMP-naïve Mφs (which has not been primed by the current MAMP/DAMP) are activated and/or recruited to phagocytose the dying and senescent priCaC, leading to the hybridization of the priCaC and Mφ with the subsequent generation of the memMφ-like THC/metCaC, probably due to defaulted degradation of the tetraploid DNA from both parental cells. As the memMφ-like THC/metCaC acquires phenotypic properties from the conventional memory macrophage (memMφ) as a result of the hybridization, its biological and immunological phenotypes are switched and begin to resemble those of the conventional memMφ. The memMφ-like THC/metCaC enters dormancy at both primary and metastatic sites and can be awakened by the priming MAMP/DAMP, just like the conventional memMφ. (II) Below the dashed line, the boxed texts describe the types of the cells that the objects represent.

We have previously proposed that cancer is a DNA repair-defective disease, and DNA repair-defective cells are predisposed to cancerization (**Box 2** item 14). As outlined in **Figure 1**, under DNA damaging conditions such as elevated free radicals resulting from stresses, chemotherapy, and radiotherapy, DNA repair-defective priCaCs are liable to senescence and/or programmed cell death (PCD), including immunogenic cell death (ICD) (**Box 2** Item 15). The senescent (93–95) and dying priCaCs secrete cytokines and other molecules whereas disintegrated cells and cancer-associated microbes (**Box 2** Item 11) at the compromised barrier tissue/organ (**Box 2** Item 12) also release immunogenic damage-associated molecular patterns (DAMPs) and pathogen-/microbe-associated molecular patterns (MAMPs) (**Box 2** Item 12). These molecules can recruit and/or prime MAMP/DAMP-naive tissue-resident Mφs and distant naïve monocytic Mφs. The recruited and/or primed Mφs engulf and/or fuse with the microbes, dying priCaCs, and their debris. However, not all engulfed and/or fused microbial and cellular components (such as their proteins and DNA) can always be degraded thoroughly. As a matter of fact, these components can often be found within the engulfing Mφs (8, 96), presumably because the components are resistant to the degrading enzyme of the Mφs, or because the Mφs are defective or deficient (8, 96). The resulting fused Mφs, therefore, possess properties of both parental priCaCs/microbes and Mφs with the concomitant phenotype switching (8, 96). (This is particularly true for cancer patients who genetically inherit DNA repair defects (**Box 2** item 14) and the resulting priCaC-Mφ hybrids cannot properly digest the excessive/tetraploid DNA from both parental cells.) At the ensuing resolution phase of the inflammation (**Box 2** Item 9) (97), some of these MAMP/DAMP-primed hybrids (Section 2) (69) differentiate into memMφ-like THCs/metCaCs (23), just like the way that conventional memMφs are generated (**Box 2** Items 8 and 9). Resembling the conventional memMφs (**Box 2** Items 1, 3, 8, and 9), the memMφ-like THCs/metCaCs can stay at the primary site as tissue-resident memMφ-like THCs/metCaCs. Alternatively, the memMφ-like THCs/metCaCs can also metastasize (i.e., migrate) directly or indirectly, i.e., relayed by the bone marrow (98, 99), to the Mφ niche at distant organs.

When an acquainted MAMP/DAMP primer appears again as a result of a second inflammation, the previously primed tissue-resident memMφ-like THCs/metCaCs are awakened and differentiate into effector progeny of the memMφ-like THCs/metCaCs via self-renewal. Similarly, the memMφ-like THCs/metCaCs located at the bone marrow and other distant peripheral organs, are also awakened and recruited to the inflammatory site (**Box 2** Item 8) (7).

Consistent with our theory, metastatic cancerous cells have been observed to be generated at the reparative resolution phage of an inflammation and the generation is mediated by leukocytes (46, 70).

According to our model, DNA repair defects inherited by or occurred to both Mφs and priCaCs are required for the generation of memMφ-like THCs/metCaCs. Indeed, defective DNA repair seems to contribute to the enhanced susceptibility of priCaCs to metastasis (3, 100, 101). The susceptibility of DNA repair-defective cells to cancerization and metastasis is probably due to two reasons. First, the DNA repair-defective priCaCs are more liable to DNA-damaging conditions, leading to more immunogenic cell death (ICD) and, subsequently, more memMφ-like THCs/metCaCs that metastasize/migrate to the distant Mφ niche (**Box 2** item 15). Second, the hybrids generated as a result of the hybridization between the priCaCs and the Mφs, have excessive/tetraploid DNA (1, 3). The excessive DNA is further processed via cellular events such as autophagy and DNA degradation, so that the hybrids are life-compatible (1). As both hybridizing priCaCs and Mφs have genetically inherited the DNA repair-defects (**Box 2** item 14), the resulting hybrids apparently cannot properly process the excessive DNA as normal cells do, leading to aberrant chromosome rearrangements and aneuploidy (i.e., genomic instability) (102) that can drive metastasis (100). Consistently, the defaulted degradation of excessive DNA occurs during or as a result of programmed cell death (PCD), leading to the genomic instability (3) such as the *MLL*-associated chromosome translocation (103).

Additional to the defaulted DNA degradation associated with cancerous cells, defaulted processing of cellular constituents of phagocytosed cells by Mφs is not rare. Defective hybridization/fusion of Mφs with other cells (including other Mφs) has also been found to contribute to the generation of multinucleated giant cells related to diseases such as tuberculosis and leprosy (96), as well as cancer-associated macrophage-like cells (1).

### 3.2 Important concept inferred from the model: types of cancerous cells at the primary tumor site and at the distant metastatic site

Like the memMφ (**Box 2** Items 8 and 9), the memMφ-like THC/metCaC can reside at the primary site as a tissue-resident. Moreover, the memMφ-like THC/metCaC can also metastasize/migrate to the Mφ niche located at distant organs. Therefore, our theory infers that at the primary site, the malignant tumor would be primarily composed of two types of cancerous cells: the priCaC and the memMφ-like THC and its effector progeny, whereas the distant metastatic tumor should only have the memMφ-like THC/metCaC and its effector progeny. As a result, the tissue-resident memMφ-like THC is immunologically more akin to and should probably be grouped with the distant memMφ-like THC/metCaC, rather than the priCaC, considering the fundamental immunological difference between the two types of cells. In this sense, the tissue-resident memMφ-like THC at the primary site should be considered as a “metCaC”, just like the distant memMφ-like THC/metCaC. The “recurrence” of the primary tumor may result from the awakening of the dormant tissue-resident memMφ-like THC. This concept may have therapeutic implication (Section 5.8).

### 3.3 Important concept inferred from the model: three distinct immune responses induced by the same primer

Three immune responses can be induced by priming MAMPs and DAMPs. (I) The primers can prime a naïve monocytic Mφ or a primer-naïve Mφ that have not previously been primed by the specific primers, subsequently causing the monocytic Mφ/Mφ to differentiate into a memMφ-like THC/metCaC following the hybridization with the priCaC. (II) Just like they do to the dormant memMφ, the primers can also awaken the previously primed dormant memMφ-like THC/metCaC. (III) The primers, if persistently and massively present, can deplete the primed memMφ-like THC/metCaC and their effector progeny in the same way as they do to the memMφ (**Box 2** Item 16).

The priming (I), awakening (II), and depleting (III) effects of the priming MAMPs and DAMPs on the memMφ-like THC/metCaC constitute the fundamental to interpret many clinical features of cancer, particularly the paradoxal effect of microbes and trauma on cancer (Sections 4 and 5). Specifically, the awakening of the memMφ-like THC/metCaC can cause the “occurrence”/recurrence of a tumor whereas the depletion of the memMφ-like THC/metCaC can induce the regression/remission of the tumor.

## 4 Interpretation of clinical manifestations of metastatic cancer with the hybrid model

### 4.1 Steps of metastatic cascade, including immunologic dormancy, dormancy, and stem-like property

As a hybrid of the priCaC and memMφ, the memMφ-like THC/metCaC has acquired biochemical molecules from the Mφ/memMφ and behaves just like the Mφ/memMφ, such as migrating across the body, evading immune surveillance (3, 104), lodging at the Mφ niche at distant organs (105), entering immunologic dormancy (105), being awakened from the dormancy by an acquainted priming MAMP/DAMP (106), and self-renewing to multiply its population for clearing local and distant microbes and damaged host cells (51).

### 4.2 Complex and paradoxal relationships between infections and primary cancer cells and metastatic cancer cells

Acute microbial infections and vaccinations, especially those acquired at young ages or before the acquisition of a cancerous disease, can reduce cancer risk (46, 107). However, for a patient who has had cancer, acute microbial infections can either facilitate metastasis (36) or, paradoxically, induce cancer regression (46, 108). On the other hand, chronic infections, particularly chronic viral infections, often promote carcinogenesis (46, 71). However, some chronic infections, especially those whose pathogeneses directly involve Mφs, such as tuberculosis and leprosy, can restrain the development of cancer (46).

As discussed above (Section 3), for a person who has cancer, the phagocytosis of priCaCs by naïve Mφs can produce primed Mφ-like THCs and memMφ-like THCs. On the other hand, for a person who does NOT have cancer, the phagocytosis of non-cancerous cells by the naïve Mφs can lead to the generation of primed conventional Mφs and memMφs. These non-cancerous, conventional, and already-primed Mφs and memMφs can clear any damaged cells, including the priCaCs, presumably without giving the cells a chance to redundantly generate the Mφ-like THCs and memMφ-like THCs. Therefore, infecting/vaccinating a person before the person acquires cancer, can reduce cancer risk.

However, once DNA repair-defective cells are converted into full-scale cancer cells (i.e., priCaCs) (**Box 2** Item 14), an unacquainted MAMP/DAMP primer of an unprecedented microbe, promotes the generation of Mφ-like THCs and memMφ-like THCs/metCaCs. (As discussed in Section 3.3, this is the priming effect of the MAMP/DAMP.) The memMφ-like THCs/metCaCs lodge dormantly at the primary tumor site and/or distant organs. These dormant memMφ-like THCs/metCaCs can be awakened later by the acquainted primer, causing cancer “occurrence”/recurrence. (As discussed in Section 3.3, this is the awakening effect of the MAMP/DAMP.)

On the other hand, a severe acute microbial infection may release a large quantity of the primer. Under this condition, Mφ-like THCs and memMφ-like THCs and their progeny can be depleted by the acquainted MAMP/DAMP primer, exhibiting cancer regression/remission. Indeed, acute microbial infections that can cause cancer regression/remission are often severe (108). (As discussed in Section 3.3, this is the depleting effect of the MAMP/DAMP.) An insufficiently severe infection may only consume a portion of the Mφ-like THCs and memMφ-like THCs, presumably causing a partial or transient regression/remission.

Chronic microbial infections, particularly those caused by intracellular viruses, are associated with increased risk of cancer (46). Among many others, a chronic inflammation continuously produces a large number of DNA-damaging free radicals, predisposing DNA repair-defective cells not only to becoming cancer cells but also to senescence and ICD, which promotes the generation of Mφ-like THCs/memMφ-like THCs (Section 3.1). (As discussed in Section 3.3, this is the priming effect of the MAMP/DAMP.) On the other hand, the chronic inflammation may also release the acquainted MAMP/DAMP primer that can awaken dormant memMφ-like THCs/metCaCs at both/either the primary site and/or distant organs. (As discussed in Section 3.3, this is the awakening effect of the MAMP/DAMP.)

However, chronic infections such as tuberculosis and leprosy that can directly damage Mφs, are likely to interfere the generation of memMφ-like THCs/metCaCs, inhibiting cancer development.

### 4.3 Impact of resection of the primary tumor on the metastatic *locus*

Surgery and other types of traumas can cause cancer recurrence or “occurrence” of a previously-unaware-of tumor at both/either primary site and/or distant organs (37). Additional to non-specific factors such as immune suppression, anesthesia, blood loss, transfusion, etc. (37), surgery at the primary site causes the release of MAMPs and DAMPs (109). The binding of the MAMPs and DAMPs to their cognate PRRs such as TLRs, awakens previously primed tissue-resident and/or distant memMφ-like THC/metCaCs, causing cancer “occurrence”/recurrence at the primary site and/or distant organs. (As discussed in Section 3.3, this is the awakening effect of the MAMP/DAMP.)

### 4.4 Oncotaxis

MetCaCs have been shown to preferably metastasize to a traumatic organ, a phenomenon that is sometimes called oncotaxis (110, 111). Damaged cells at the traumatic organ release MAMPs/DAMPs as well as cytokines. These molecules activate and recruit memMφ-like THCs/metCaCs, which have previously been primed by the same DAMPs/MAPMs, from the distant Mφ niche to resolve the trauma; importantly, the awakening of the memMφs/memMφ-like THCs depends on the chemical structure of the MAMP/DAMP primers, irrespective of which species, strains or types of the microbes/damaged host cells that release the MAMPs/DAMPs (**Box 2** Items 12 and 13). (As discussed in Section 3.3, the oncotaxis is due to the awakening effect of the MAMP/DAMP.)

### 4.5 “Spontaneous” and Coley’s toxin-induced regression of primary and metastatic cancer

Occasionally, a tumor at the primary and/or metastatic sites can “spontaneously” regress following infectious diseases (70, 108, 112, 113). The “spontaneous” regression usually occurs following a severe infection (70, 108).

Coley’s toxin can also cause regression of cancer. However, to have a long-term, rather than a transient, regression, Coley’s toxin is often required to be administered daily or every other day for months (70, 112, 113). Otherwise, the regressed tumor can recur (70, 112, 113).

The severe infectious disease and repeated administration of high doses of Coley’s toxin suggest that depletion of memMφ-like THCs/metCaCs contribute to the regression of cancer; the depletion of memMφs/memMφ-like THCs requires high doses of acquainted DAMPs/MAPMs (**Box 2** Item 16). This is consistent with the observation that cancer can recur if the exposure of patients to either microbes or Coley’s toxin is immaturely ceased (70, 112, 113). This is also aligned with the observation that fever is important for Coley’s toxin-induced regression (108) as well as the observation that the regression of the tumor can occur shortly after an infection, even within hours (46, 113). Both fever (108) and quick regression following an infection are indicative of innate immune responses (46, 113), including those associated with Mφs/Mφ-like THCs and memMφs/memMφ-like THCs. Indeed, the “spontaneous” regression involves the specific biding of MAMPs/DAMPs to their cognate PRRs (such as TLRs) of innate immune cells (108). Therefore, as discussed in Section 3.3, the “spontaneous” and Coley’s toxin-induced regression is the depleting effect of the MAMP/DAMP.

Additional to depleting Mφs, MAMPs/DAMPs can also induce tolerance, paralysis and other immune responses of the Mφs, depending on the dose of the MAMPs/DAMPs (**Box 2** Item 16) (114). Therefore, it is possible that these immune responses can also contribute to the persistent, transient or partial regression of cancer.

### 4.6 Modulation of primary and metastatic cancers by gut microbiota

The gut microbiome is known to modulate the tumor microenvironment (81). The composing commensal and pathogenic microbes of the microbiome can directly secret MAMPs and indirectly release DAMPs from the damaged cells, to prime naïve monocytic Mφs and MAMP-naive Mφs. Alternatively, the MAMPs/DAMPs can also awaken dormant memMφs primed previously (115–117). Being like Mφs, Mφ-like THCs and memMφ-like THCs/metCaCs can also undergo the same process by the MAMPs/DAMPs (118, 119). Therefore, the microbiome can modulate the primary and metastatic tumors.

A related issue is that barrier tissues/organs (such as the skin, respiratory tract, gastrointestinal tract, and urogenital tract) and barrier-connected tissues/organs (such as exocrine glands connected to the skin and tracts) are much more prone to cancer than non-barrier tissue/organs such as the heart and spleen; the latter is less likely to be exposed to microbes.

## 5 Therapeutic strategies and associated issues

For the past several decades, a large number of immune therapeutic strategies have been developed, including vaccination, CAR-T, and checkpoint inhibition (120). These innovative and revolutionizing therapies have been discussed throughout the literature. As a result, they are not re-iterated here. Instead, we only discuss potential therapeutic strategies (such as Coley’s toxin-like agents) that are closely related to the current proposed theory, particularly those targeting the memMφ-like THC/metCaC.

### 5.1 Specific depletion of memory macrophage-like tumor hybrid cells/metastatic cancer cells and their effector progeny with microbes/microbial extracts

Depletion of memory immune cells, such as T cells and Mφs, has been explored to treat several chronic inflammatory diseases (121–123). As Mφs are deeply involved in the metastatic cascade (46), depletion of tumor-associated macrophages (TAMs) has also shown positive therapeutic potential. Although most of non-specific Mφ depleters can block metastasis by depleting active Mφs such as TAMs at both primary and metastatic sites, they often cannot eliminate dormant memMφs/metCaCs (64, 69, 122, 124). Additionally, considering the wide immunological role of Mφs, non-specific depletion, particularly long-term non-specific depletion, of the Mφs may lead to serious side-effects. As a result, Mφ depleters that specifically deplete memMφ-like THCs/metCaCs, are more likely to be therapeutically required.

One of the possible specific depleters is composed of priming commensal and pathogenic microbes or their extracts such as Coley’s toxin. Indeed, additional to Coley’s toxin, a number of other microbe/microbial extract preparations, such as bacillus Calmette-Guerin (BCG) and lipopolysaccharides (LPSs), have been used to treat cancer experimentally and clinically (108, 112, 113, 125).

An advantage of using a whole microbe or its extract is that it includes all types of MAMPs/DAMPs of the microbe. Therefore, many or even all clones of the memMφ-like THC/metCaC primed by various types of chemically-structured priming MAMPs/DAMPs can be depleted by a single microbial preparation, contrasting to depleters that only target one specific chemically-structured MAMP/DAMP and can only deplete limited clones of the memMφ-like THC/metCaC (See next Section 5.2).

The microbiome is often distinctive from persons to persons, organs to organs of the same person, and regions to regions of the same organ (such as different segments of the bowel); the latter is known as “compartmentalization” (**Box 2** Item 13). Moreover, MAMPs released by different microbes may not necessarily be identical and can be overlapping or even completely different. For example, Mφs primed by a fungus do not always kill any bacteria (126, 127). As a result, patient-, organ-, and compartment-specific Coley’s toxin-like microbes/microbial extracts may be required for patient- or even tumor-specific treatment. On the other hand, influenza-trained Mφs are found to be both tissue-specific and phagocytic and cytotoxic against metastatic lung cancer (128).

To have a patient- or tumor-specific anti-tumor microbe/microbial extract, analyzing the local microbiome and identifying its composing microbes, including commensals and pathogenic and historic pathogenic microbes, in the vicinity of the tumor is probably beneficial.

A preparation composed of a broad spectrum of microbes, including bacterial, viral, fungal, and/or parasitical extracts (129), is indicated as a universal memMφ-like THC/metCaC depleter to treat the metastatic cancer of unknown primary origin or a metastatic cancer that is not responsive to a specific microbial depleter. However, side effects, such as immune paralysis of the conventional immunity against these microbes should be evaluated.

Intentionally awakening the dormant memMφ-like THC/metCaC of a patient whose disease is inactive and undetectable for the purpose of eliminating all dormant hybrids, is probably not necessary.

Side-effects of the Mφ-depletion strategy should also be evaluated. For example, what might occur to patients if they contract the same infection again (a third infection)? Would the patients tolerate the microbes? Would the tolerated microbes can grow un-controllably and cause harm to the patients? These issues may need to be studied in clinical translation.

### 5.2 Specific depletion of memory macrophage-like tumor hybrid cells and their effector progeny by targeting at MAMPs/DAMPs, receptors of the MAMPs/DAMPs, and downstream signaling pathways

According to our theory, a memMφ-like THC/metCaC clone and its effector progeny can specifically be depleted by the MAMP/DAMP that has previously primed them. Likewise, a chemical targeting a primer-specific PRR and its downstream signaling molecules can also deplete/inhibit a specific memMφ-like THC/metCaC clone (130). However, the caveat is that a depleter targeting a specific MAMP/DAMP, PRR or signaling molecule can only eliminate the specific memMφ-like THC/metCaC clone that has previously been primed by the MAMP/DAMP (**Box 2** Items 12 and 13), contrasting to a whole microbe or microbial extract preparation that is composed of all MAMPs of the microbe and can deplete all memMφ-like THC/metCaC clones.

### 5.3 Restrain dormant memory macrophage-like tumor hybrid cells/metastatic cancer cells to prevent their awakening/recurrence

The awakening/activation of monocytic Mφs can be blocked by the inhibitory PRR (iPRR), such as LILRB1, LILRB3, Siglec 10, and SIRL-1, which blocks the specific binding of a MAMP/DAMP to its cognate PRR (131). Therefore, the iPRR can also potentially be explored to prevent the awakening/recurrence of the dormant memMφ-like THC/metCaC. This anti-cancer strategy probably requires a long-term administration of the iPRR, particularly during inflammatory events.

### 5.4 Anti-inflammatory and macrophage-depleting approaches to inhibit recurrence of primary and metastatic tumors

As discussed (**Figure 1**), the memMφ-like THC/metCaC is generated as a result of a sterile and/or non-sterile inflammation. Therefore, anti-inflammatory or other anti-immune drugs, such as NSAIDs and antibiotics, may prevent the recurrence of both primary and metastatic tumors by blocking the release of MAMPs/DAMPs and the subsequent awakening/recurrence of the dormant tissue-resident and distant memMφ-like THCs/metCaCs. Consistently, immunosuppressive dexamethasone contains the invasiveness of cancer (132).

However, whether resolution-associated molecular patterns (RAMPs) and specialized pro-resolving mediators (SPMs) (68) can promote or inhibit metastasis remains to be investigated.

### 5.5 Combination of chemotherapy, radiotherapy and other cytotoxic therapies with anti-inflammatory/anti-immune, antibiotic, and Mφ-depleting drugs

Chemotherapy can promote metastasis (133). The metastasis-promoting effect of chemotherapeutic anti-cancer drugs, according to the current theory, results from the release of MAMPs/DAMPs, which lead to the generation of the memMφ-like THC/metCaC and/or the subsequently awakening of the dormant memMφ-like THC/metCaC located at the primary site and/or distant niche. Therefore, co-administration of antibiotics and anti-inflammatory drugs (such as NSAIDs) should inhibit the metastasis-promoting effect of the cytotoxic therapy. Mφ depleters should have a similar effect by inhibiting the generation of the metCaC/memMφ-like THC. However, it remains to be tested whether these strategies are indeed true.

### 5.6 Peri-surgery administration of antibiotic, anti-inflammatory, and anti-immune drugs

As discussed above (Section 4.3), surgical excision of the primary tumor may release MAMPs/DAMPs into the blood circulation, not only inducing the generation of memMφ-like THCs/metCaCs but also awakening dormant memMφ-like THCs/metCaCs. Therefore, peri-operative administration of antibiotic, anti-inflammatory (such as NSAIDs and dexamethasone) or Mφ-depleting/interfering drugs may reduce the occurrence/recurrence of metastasis (109, 122, 134). β-blockers that interfere with the infiltration of Mφs have a similar effect and may be administered as well (134). However, their side effects on healing process may need to be balanced.

Additionally, the edge of excision should be as far away as possible from the primary tumor to prevent the release of anatomic site-specific MAMPs/DAMPs (**Box 2** Item 13), which can awake dormant memMφ-like THCs/metCaCs.

### 5.7 Distinct strategies required for *in situ* primary tumor and metastatic tumors

As discussed above (Section 3.2), the primary tumor primarily consists of two types of cancerous cells, the priCaCs and tissue-resident Mφ-like THCs/memMφ-like THCs. These two types of cells are entirely distinctive entities from the immunological perspective. The tissue-resident Mφ-like THCs/memMφ-like THCs should immunologically be grouped with the “central”/distant Mφ-like THCs/memMφ-like THCs (Section 3.2). Consistently, therapies targeting the *in situ* priCaCs may have an opposite effect on the memMφ-like THCs/metCaCs and *vice versa* (135, 136). Therefore, distinct strategies are likely to be required for the primary tumor and the metastatic tumor.

As priCaCs at the inflammatory primary tumor site constantly generates Mφ-like THCs and memMφ-like THCs/metCaCs, whenever doable, excision of the primary tumor is probably necessary irrespective of the clinical stage into which a patient is categorized. Alternative, a therapy that can simultaneously target both local (primary) and systemic (metastatic) tumors may be applied (70).

### 5.8 Monitoring the memory macrophage-like tumor hybrid cells/metastatic cancer cell during immune depletion therapy

Coley’s toxin is usually required to be administered for months for a long-term effect. Ceasing the administration of the toxin immaturely can lead to the recurrence of the metastatic tumor (70, 112, 113). This is likely to be applicable to other Mφ-depleting agents. Therefore, monitoring the residual memMφ-like THC/metCaC and its progeny in the blood should help to determine when the administration of the Mφ-depleting therapy should be terminated. Measuring the Mφ-like THC- and memMφ-like THC/metCaC-specific genetic (such as oncogenes), epigenetic, and/or other cellular markers can be a feasible way to monitor if the memMφ-like THC/metCaC is depleted. Techniques analyzing the profile of cancer cell surface proteins and MAMPs/DAMPs have been developed (137).

### 5.9 Staggered outgrowth of dormant malignant hybrid

As the chronic inflammation at the primary tumor site is likely to be episodically/periodically alleviated and intensified as a result of the fluctuation of DNA damage, distinctive MAMPs/DAMPs may be released, causing the generation, as well as the awakening, of different batches/clones of memMφ-like THCs/metCaCs. Therefore, it is likely that not all of the dormant memMφ-like THC/metCaC clones can be awakened by a single traumatic or infectious event (1). Rather, the dormant memMφ-like THCs/metCaCs generated at the distinct episodes of the inflammation are likely to be periodically awakened upon repeated traumatic and infectious stresses (138), and staggered outgrowth of distinct clones of the memMφ-like THCs/metCaCs may occur (139), at the same or different metastatic organs (such as lung and brain metastases of the breast cancer). Therefore, treatment against metastasis might be a long-term effort with periodic administration of anti-metastatic therapies.

### 5.10 The relationship between cytotoxic T cells and macrophages/macrophage-like tumor hybrid cells

The number of T cells infiltrated within tumors is positively correlated with the prognosis of cancer patients (140). However, relationships between T cells and conventional Mφs are much more complex. Contrasting to pro-inflammatory and anti-tumor M1 Mφs (**Box 2** Item 2) or M1-like TAMs (141) which present antigens to T cells and interact with T cells for many other immune-regulatory roles, M2 Mφs (**Box 2** Item 2) or M2-like TAMs induce T cell exhaustion (142–145), presumably with the concurrent generation of memory T cells (144) during the reparative resolution phase of an inflammation. Therefore, quantitatively, conventional Mφs and T cells are sometimes inversely related, which is indicative of an immune-regulatory relationship. T cell elevation suggests that Mφs, including memMφs, could be suppressed or enter quiescence/dormancy.

Considering that memMφ-like THCs/metCaCs resemble conventional memMφs, their relationship with T cells is likely to be immune-regulatory as well. Indeed, T cells inhibit the outgrowth of memMφ-like THCs/metCaCs by cytostatic rather than cytotoxic effects (146). Consistently, T cells restrain memMφ-like THCs/metCaCs from awakening from dormancy, rather than kill them (139). T cell depletion, on the other hand, leads to the outgrowth of dormant memMφ-like THCs/metCaCs (71). Moreover, recent studies have shown that the infiltration of T cells only occurs at the late stage of tumor regression whereas a group of specific Mφs (MHCII^+^CD163^-^) are closely associated with the regression (147). These observations are consistent with the theory that memMφ-like THCs/metCaCs resemble memMφs.

### 5.11 Prevention of the occurrence or recurrence of metastatic cancer cells via the induction of inert immune states

So far, we have primarily discussed how to eliminate the memMφ-like THC/metCaC by depleting it. However, like that of the memMφ (**Box 2** Item 16), the recurrence/awakening of the memMφ-like THC/metCaC can be suppressed via the induction of the inert state, similar to immune anergy, paralysis or tolerance of the Mφ/memMφ.

A preparation composed of specific microbes or their extracts may be administered as a prevent strategy for individuals who are genetically susceptible to a specific type of cancer.

## 6 Unanswered questions related to the theory

The research on the innate immune memory (trained innate immunity) itself is at the teenage stage. Consequently, a number of questions regarding the current proposed theory wait to be answered.

### 6.1 Where does the memory macrophage-like tumor hybrid cell/metastatic cancer cell acquire the ability to metastasize between peripheral organs or from peripheral organs to the bone?

Studies have shown that priCaCs may acquire the ability to metastasis from conventional Mφs following their hybridization with the Mφs (8, 9, 46, 47). However, conventional Mφs themselves have not been found to be able to migrate from the inflammatory periphery to other peripheral organs (such as the lungs, liver, and brain) or the bone (central) and lodge there (10). On the other hand, monocytes and HSPCs at the bone marrow have been found to have memory from a previous infection at the periphery (10). Additionally, intestinal bacteria can train alveolar Mφs to defend the lungs against a future viral infection (148). Therefore, where do these memory cells come from and how are they generated? Currently, memory monocytes and memory HSPCs at the bone marrow and other distant organs, are considered to be trained by circulating microbes or their constituents (10, 148), rather than a DIRECT migration and lodging of the memory cells from the inflammatory periphery. If this is the case, why the circulating microbes or their constituents only train the memory monocytes and memory HSPCs at some specific organs (i.e., the bone and lungs as in the cases above), but not those at other organs. Moreover, memory immune cells are often generated as a result of an inflammation and at the late stage of the inflammation. How then, can the bone marrow monocytes and HSPCs acquire memory without an inflammation at the bone marrow?

On the other hand, Mφs (not monocytes) can be found in the blood among patients having viral infections, cancer, and autoimmune diseases (43–45), suggesting that Mφs are released from the inflammatory periphery and traffic through the blood circulation. Moreover, the Mφs can be present in the blood for weeks and months, rather than days, after the infection is resolved, which is indictive of innate immune memory (44). To find whether blood-circulating Mφs belong to memMφs or their effector progeny and whether they can lodge at the bone and other distant organs, analyses of the genetic and chromosomal alterations of the Mφs/memMφs might be of value, considering that genotypes are less vulnerable to the changing microenvironment than phenotypes. Following the phagocytosis of cells undergoing ICD, monocytes/Mφs are required to digest and process the engulfed excessive DNA (1, 3). Therefore, cell- and clone-specific genetic and chromosomal alterations are expected (149, 150). As a result, comparing the alterations of the monocytes/Mφs from the inflammatory periphery, blood, and bone might clarify whether memMφs can communicate among these organs, like memMφ-like THCs/metCaCs do.

### 6.2 Can a metastatic cancer cell undergo further metastasis?

Several studies have shown that a metCaC located at one (the first) distant organ can further metastasize to another (the second) distant site, a phenomenon called metastasis-to-metastasis. However, the opposite has also been observed (98, 99, 151).

According to the current theory, whether a metCaC lodging at the first site can metastasize again to the second organ depends on the following conditions. The second organ depletes its tissue-resident Mφs, demanding supplements of Mφs and Mφ-like THCs (effector progeny of awakened memMφs and memMφ-like THCs/metCaCs) from across the body. Moreover, the priming MAMP/DAMP released from the second metastatic organ and the primer that has preciously primed the memMφs and memMφ-like THCs/metCaCs, should share the same chemical structure. Additionally, cytokines released from the second organ should match cytokine receptors on the memMφ-like THCs/metCaCs located at the first organ.

The metastasis-to-metastasis (from the first metastatic site to the second metastatic site) should be differentiated from the metastasis of multiple organs from the same primary tumor.

### 6.3 Do all memory macrophage-like tumor hybrid cells/metastatic cancer cells have the same proportion of cell contents from hybridizing primary cancer cells and macrophages?

The Mφ-/memMφ-like THC can be generated via several ways, including fusion (152), trogocytosis (55), exchanging cellular contents via extracellular vesicles (153), undergoing cell-in-cell (including entosis, emperipolesis, and cell cannibalism) (154), efferocytosis (154), etc. Therefore, the respective proportions of the priCaC and Mφ that constitute the hybrid, are likely to differ from one hybrid to another (5). This implicates that the immunological and biological behaviors of each memMφ-like THC/metCaC may differ, an issue that can be relevant to patient management.

### 6.4 What is the type of the metastatic cancer cell that metastasizes to the lymph node?

We have primarily discussed the hematogenous metCaC, which is a hybrid of the priCaC and Mφ. However, the cancer cell also frequently metastasizes to the lymph node, conceivably via the lymphatic vessel. Previous studies have found that the metastatic cancer cell at the lymph node expresses surface markers of the memory lymphocyte (**Table 1**) (155). Like the memory lymphocyte, the metastatic cancer cell metastasizing to the lymph node, is also located around the high endothelial venule (HEV) (156). Additionally, the hybridization between the priCaC and the lymphocyte, specifically, transferring of immune regulatory molecules (CTLA4 and Tim3) from the tumor-infiltrating lymphocyte to the colon cancer cell via trogocytosis, has also been reported (55). However, only group 2 innate lymphoid cells (ILCs) seem to possess the phagocytosing property (157) and are also the major memory ILCs (158, 159). Therefore, the possibility that the lymphogenous metastatic cancer cell is a memory lymphocyte-like cancerous hybrid cannot be excluded.

### 6.5 Can the theory benefit the study on autoimmune diseases, dementia, and other diseases?

Innate immune memory has recently been recognized to contribute to the pathogenesis and treatment of a number of diseases, including autoimmune diseases (3), organ/tissue transplantation (160, 161), vaccination (129), cardiovascular disorders (10, 48), Alzheimer disease (10, 48, 77), etc. Likewise, depletion of Mφs has also been explored to treat these diseases (121–123). Non-specific Mφ-depleting strategies can potentially reduce the number of pathogenic Mφs, specifically the Mφs/memMφs that have been primed by autologous DAMPs, and thereby relieve the diseased conditions of the patients. On the other hand, it is not known what the immune system would respond to a previously encountered microbe after the MAMP-primed, microbe-specific Mφs/memMφs have been depleted. Would the immune system to the body tolerate the re-encountered microbe, which could potentially cause microbe-induced damage of the body, or the immune system would *de novo* generate new Mφs/memMφs again just like it does to other newly encountered microbe? Therefore, studies on metCaCs and the treatment of the metCaCs should also benefit patients suffering from other diseases.

## 7 Summaries

We have proposed that the hematogenous metCaC is a memMφ-like THC between the priCaC and memMφ. The memMφ-like THC/metCaC is generated as a result of immunogenic cell death of the priCaC, an intrinsic event of an inflammatory response, and the subsequent defaulted processing of the excessive/tetraploid DNA due to defective DNA repair of both cells. Like the conventional memMφ, the memMφ-like THC/metCaC can enter dormancy within the Mφ niche at both primary and distant sites, and be awakened from the dormancy by acquainted priming MAMPs and DAMPs, causing “occurrence”/recurrence of the metastatic tumor. The memMφ-like THC/metCaC can also be depleted/exhausted by excessive quantities of the MAMPs and DAMPs, paradoxically leading to “spontaneous” and Coley’s toxin-induced regression of the tumor (Section 4).

Considering the complexity of cancer, the current theory does not intend to address all aspects of the hematogenous metastasis of solid tumors, let alone hematological neoplasia. For example, additional to Mφs, other types of cells can also fuse with cancer cells (102). The contribution of these cancerous hybrids to the tumors, remains to be studied. Additionally, for renal cell carcinoma (RCC), surgical excision of the primary tumor may sometimes lead to the regression, rather than recurrence, of the metastatic counterpart (162). It is not known whether this is because the kidney is generally considered as a non-barrier organ, which is minimally populated with commensal microbes, or because the immune contexture at the primary site does not support the generation and storage of Mφs, similar to those of cytotoxic T cells (163). Therefore, the current theory is only intended to provide a possibility, from another perspective, to address the hematogenous metastasis of some solid tumors, particularly the types of solid tumors whose prCaCs are infected by microbes and are subsequently phagocytosed/fused/hybridized with priming microbe-naïve Mφs.

## Author’s note

This piece of work is part of CJ's master degree thesis project.

## Data availability statement

The original contributions presented in the study are included in the article/supplementary material. Further inquiries can be directed to the corresponding author.

## Author contributions

JW: Conceptualization, Data curation, Funding acquisition, Supervision, Writing – original draft. CJ: Data curation, Validation, Writing – review & editing.
